# The complete mitochondrial genome of *Hapalogenys analis* (Perciformes, Haemulidea) except for control region, obtained by whole genome sequencing

**DOI:** 10.1080/23802359.2020.1788463

**Published:** 2020-07-14

**Authors:** Yunya Zheng, Linxiao Feng, Haoyu Liu, Riyu Song, Shengyong Xu, Huilai Shi, Tianxiang Gao

**Affiliations:** a Fisheries College, Zhejiang Ocean University, Zhoushan, Zhejiang, China; b Zhejiang Province Key Lab of Mariculture and Enhancement, Marine Fisheries Research Institute of Zhejiang, Zhoushan, Zhejiang, China

**Keywords:** Haemulidea, mitogenome, *Hapalogenys anails*, next-generation sequencing

## Abstract

In this study, we obtained the complete mitochondrial genome of *Hapalogenys analis* using whole genome sequencing. With the exception for control region, this mitochondrial genome, consisting of 16,355 base pairs (bp), contains 13 protein-coding genes (PCGs), 2 ribosomal RNAs (rRNAs), and 21 transfer RNAs (tRNAs). This mitochondrial genome also lacks a tRNA-Pro gene after tRNA-Thr gene. The overall base composition shows 25.45% of T, 29.73% of C, 28.68% of A and 16.14% of G, with a slight A + T rich feature (54.13%). Sanger sequencing is needed to confirm the accuracy of control region, as well as the lack of tRNA-Pro gene. The mitogenome data provides useful genetic markers for the studies on the molecular identification, population genetics, phylogenetic analysis and conservation genetics.

The *Hapalogenys anails* is an important economic species distributed mainly in the northwestern Pacific, including the coastal waters of southern China and Japan (Xu et al. 2010). This species is a kind of demersal fish living in the water depth of 30–50 m, and often occurs in artificial reef areas (Xu et al. 2010). To date, the taxonomic status of genus *Hapalogenys* is controversial and problematic (Wei et al. 2014). However, only limited genetic information have been published for *H. anails*. In this study, the next-generation sequencing technology is used to determine the complete mitochondrial genome of *H. anails*. The structure of mitogenome is compared and analyzed, and some gene markers are further used for the phylogenetic status of this species.

The sample of *H. anails* was collected from the Xixuan Island coastal waters of Zhoushan (29.54°N, 122.19°E), China in September 2019. The examined specimen was preserved at Fisheries Ecology and Biodiversity Laboratory in Zhejiang Ocean University under specimen accession no. ZJOU-03742. The genomic DNA was extracted from dorsal-lateral muscles (30 mg) using Rapid Animal Genomic DNA Isolation Kit (Sangon Biotech Co., Ltd., Shanghai, CN). A genomic library was established and followed by the next-generation sequencing. Whole genome sequencing (sequencing depth 50X) was conducted using Illumina Hiseq4000 platform with the sequencing insertion of 350-bp. Quality check for sequencing data was done by FastQC (Andrews 2010) and the filtered clean data were assembled and mapped to complete mitogenome sequence using NOVOPlasty v3.7.2 (Dierckxsens et al. 2017). Subsequently, the assembled sequence was annotated using the online Mitochondrial Genome Database of Fish server (Iwasaki et al. 2013) and the MITOS Web Server (Bernt et al. 2013).

The complete mitogenome was assembled using NOVOPlasty software initially. However, with the lack of tRNA-Pro, we doubted the accuracy of control region sequences. As a result, the final sequence except for tRNA-Pro and control region has been deposited in GenBank with accession number MT561534. The mitochondrial genome of *H. anails* in this study (16,355 bp in length) consists of 13 protein-coding genes, 21 tRNA genes, and 2 rRNA genes. The arrangement of all genes is identical to those of most vertebrates (Wang et al. 2008; Chen 2013; Chiang et al. 2013). Most of the genes are encoded on the heavy strand (H-strand), except for the eight tRNA genes (-Gln, -Ala, -Asn, -Cys,-Tyr, -Ser, and -Glu) and one protein-coding gene (ND6). The overall base composition is 25.45% for T, 29.73% for C, 28.68% for A, and 16.14% for G, with a slight A + T-rich feature (54.13%). Except for COI starting with GTG, the remaining 12 protein-coding genes start with ATG. It is important to note that some of the protein-coding genes (6 of 13 genes) are inferred to terminate with an incomplete stop codon (COII, COIII, ND2, ND3, ND4 and Cyt *b*), with six (COI, ATPase8, ATPase6, ND1, ND4L and ND5) sharing TAA and one (ND6) using TAG as a stop codon, respectively. These features are common among vertebrate mitochondrial genome, and TAA is supposed to be appeared via posttranscriptional polyadenylation (Ojala et al. 1981). The longest one is ND5 gene (1842 bp) among protein-coding genes, whereas the shortest is ATPase 8 gene (168 bp). The two ribosomal RNA genes, 12S rRNA (953 bp) and 16S rRNA (1697 bp), are located between tRNA^Phe^ and tRNA^Leu^.

Phylogenetic relationships were constructed using neighbour-joining (NJ) algorithm implemented in MEGA 6 (Tamura et al. 2013) among 6 species of family Haemulidea based on 12 H-strand mitochondrial protein-coding genes (Figure 1). This phylogenetic tree showed that *H. anails* has a relatively close relationship with *H. nigripinnis*. The information of the mitogenome will be useful for future phylogenetic studies and specimen identification of Haemulidea species.

**Figure 1.**
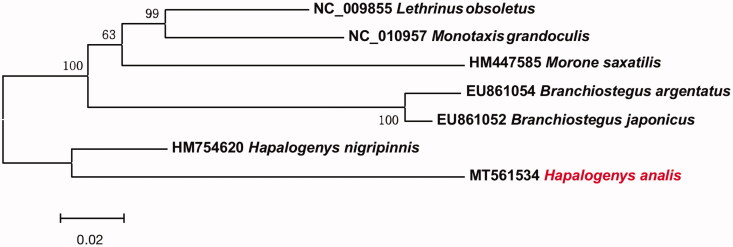
Neighbor-joining (NJ) topology for 6 species of family Haemulidae based on 12 H-strand mitochondrial protein-coding genes.

## Data Availability

The data that support the findings of this study is openly available in GenBank of NCBI at https://www.ncbi.nlm.nih.gov under accession number MT561534.
